# Reef-building corals farm and feed on their photosynthetic symbionts

**DOI:** 10.1038/s41586-023-06442-5

**Published:** 2023-08-23

**Authors:** Jörg Wiedenmann, Cecilia D’Angelo, M. Loreto Mardones, Shona Moore, Cassandra E. Benkwitt, Nicholas A. J. Graham, Bastian Hambach, Paul A. Wilson, James Vanstone, Gal Eyal, Or Ben-Zvi, Yossi Loya, Amatzia Genin

**Affiliations:** 1 The Coral Reef Laboratory, Ocean and Earth Science, University of Southampton, Southampton, UK; 2 Lancaster Environment Centre, Lancaster University, Lancaster, UK; 3 Ocean and Earth Science, University of Southampton, Southampton, UK; 4 The Mina & Everard Goodman Faculty of Life Sciences, Bar Ilan University, Ramat Gan, Israel; 5 Marine Palaeoecology Laboratory, School of Biological Sciences, The University of Queensland, Brisbane, Queensland Australia; 6 Scripps Institution of Oceanography, University of California, San Diego, La Jolla, CA USA; 7 School of Zoology, The George S. Wise Faculty of Life Sciences, Tel Aviv University, Tel Aviv, Israel; 8 Department of Ecology, Evolution & Behavior, Hebrew University of Jerusalem, Jerusalem, Israel; 9 The Interuniversity Institute for Marine Sciences, Eilat, Israel

**Keywords:** Marine biology, Animal physiology

## Abstract

Coral reefs are highly diverse ecosystems that thrive in nutrient-poor waters, a phenomenon frequently referred to as the Darwin paradox^1^. The energy demand of coral animal hosts can often be fully met by the excess production of carbon-rich photosynthates by their algal symbionts^2,3^. However, the understanding of mechanisms that enable corals to acquire the vital nutrients nitrogen and phosphorus from their symbionts is incomplete^4–9^. Here we show, through a series of long-term experiments, that the uptake of dissolved inorganic nitrogen and phosphorus by the symbionts alone is sufficient to sustain rapid coral growth. Next, considering the nitrogen and phosphorus budgets of host and symbionts, we identify that these nutrients are gathered through symbiont ‘farming’ and are translocated to the host by digestion of excess symbiont cells. Finally, we use a large-scale natural experiment in which seabirds fertilize some reefs but not others, to show that the efficient utilization of dissolved inorganic nutrients by symbiotic corals established in our laboratory experiments has the potential to enhance coral growth in the wild at the ecosystem level. Feeding on symbionts enables coral animals to tap into an important nutrient pool and helps to explain the evolutionary and ecological success of symbiotic corals in nutrient-limited waters.

## Abstract (web summary)

Long-term experiments show that corals acquire dissolved inorganic nitrogen and phosphorus by feeding on symbiont cells, which provide essential nutrients enabling their success in nutrient-poor waters.

## Main

Symbiotic corals function as mixotrophs in which the metabolic carbon demand of the animal host can often be met by the translocation of carbon-rich photosynthetic products from their dinoflagellate symbionts^2,3^. Although this carbon transfer sustains the host’s energy production, it cannot promote its growth^10^. Instead, the host is thought to take up nitrogen (N) and phosphorus (P) in a favourable stoichiometry required to produce the essential building blocks for growth and reproduction mostly by feeding on particulate or dissolved organic material, including plankton and dissolved free amino acids^11–13^. The symbionts benefit from host heterotrophy by recycling N- and P-rich excretion products of the host metabolism that they can then use to promote their own growth^11,12,14^. Retaining these valuable compounds within the symbiotic association is considered the other main function of the photosynthetic partner^10^.

**Extended Data Fig. 1:**
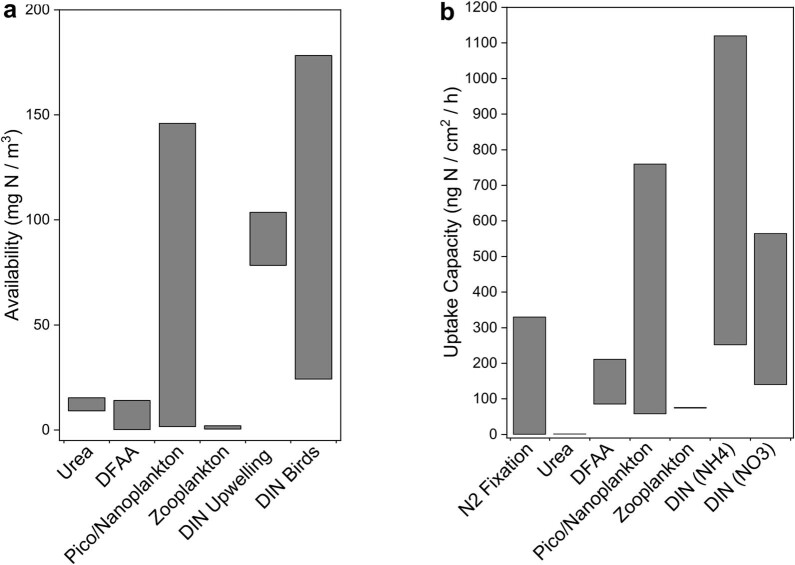
Environmental nitrogen supply to symbiotic corals. (**a**) Examples of the availability of N in different chemical forms in reef waters that can be taken up by symbiotic corals. (**b**) Capacity of symbiotic coral to take up nitrogen in different chemical forms including dissolved free amino acids (DFAA). Bars represent the range of nitrogen availability and uptake capacity defined by the upper and lower boundary values deduced from the published literature. Relevant references and unit transformation measures are summarised in Extended Data Tables 1 and 2. Dissolved inorganic N (DIN) values provided in (**a**) are for (i) upwelling areas in which coral dominance is increased by 44% compared to comparable habitats with less exposure to internal wave driven upwelling and for (ii) reef waters in which the nutrient provision by seabirds results in elevated DIN values (up to >90% nitrate) and 3-fold increase growth *Acropora formosa*.

Coral animals have the capacity to incorporate some ammonium (NH_4_^+^) directly^15,16^. However, this direct uptake pathway is quantitively trivial compared to the 14–23 times higher NH_4_^+^ assimilation rates of their symbionts^16^. By contrast, coral hosts cannot directly assimilate nitrate (NO_3_) because the animal tissue lacks the required enzymes^15,17^. Therefore, NO_3_ uptake and assimilation proceeds exclusively through the symbionts^18^. The same applies to phosphorus in its dissolved inorganic form (PO_4_)^11^. The extent to which N and P acquisition by the symbionts contributes to host growth is unclear and knowledge of nutrient partitioning remains incomplete. Previous studies using isolated symbionts suggested that only small quantities of N in the form of amino acids are released from the symbiont cells and might be available to the host^5,7–9^. More recently, nanoscale secondary ion mass spectrometry (NanoSIMS) experiments have visualized the translocation of ^15^N from the symbiont, the prominent site of N uptake, to the host^16,18^. Also, the translocation of substantial amounts of N from the symbiont to the host has been observed in an *Acropora* coral in the wild^6^. Furthermore, recent studies indicate that considerable amounts of N-rich amino acids in the host tissue originate from the symbionts^16,19–21^. At present, there is no evidence for the symbiont-to-host transfer of phosphorus^11^. In fact, symbionts are considered a phosphorus sink within the symbiosis^4^. Therefore, current knowledge cannot explain host growth-promoting effects of dissolved inorganic N and P described by several studies of corals in experimental settings and in the natural environment^22–29^. Consequently, a key mechanism controlling the productivity of the world’s coral reefs remains insufficiently understood; a fact that is of particular concern because dissolved inorganic nutrients can represent locally or temporarily the most significant sources of N or P in otherwise nutrient-poor tropical waters (Extended Data Fig. 1).

## Access to dissolved inorganic N and P

Even in nutrient-poor ocean basins, coral reefs and their surrounding waters can be replenished regularly with dissolved inorganic N and P from deeper, nutrient-rich water through upwelling, internal waves and vertical mixing^22,30–34^. In such oligotrophic waterbodies, nutrients tend to be taken up rapidly by primary producers and therefore enter reefs often in the form of phytoplankton^35,36^. However, sponges and other filter feeders that live in and on the reef framework graze on this plankton and remineralize the organic N and P^29,36,37^. The re-release of the nutrients in their inorganic form in the intimate vicinity of the corals can represent the most significant import pathway for new nutrients to reef^36,37^. Further dissolved inorganic nutrients can be introduced into reef systems from terrestrial sources and by excretion products of migrating fish and seabirds (guano)^28,38–40^. The deposition of seabird guano, specifically, can introduce significant amounts of nitrogen and phosphorus to reef systems^28,38–40^. Indeed, the amount of N available in dissolved inorganic form, mostly nitrate, in reefs close to seabird colonies can be about 90-fold higher than the amount available to corals in the form of zooplankton in reefs elsewhere (Extended Data Fig. 1 and Extended Data Tables 1 and 2).

Supply of dissolved inorganic N and P has two potential consequences for symbiotic corals. First, nutrients become directly available to the symbionts^15,22,28,32,41,42^. Second, nutrients can increase the productivity of the wider natural or experimental ecosystem^20,31^, yielding increased particulate organic N and P that may serve as food to the coral animal host^20^. Therefore, quantifying the extent to which each partner in the coral–dinoflagellate symbiosis benefits from increased nutrient levels is notoriously difficult. Here, we reveal a mechanism by which coral animals profit from dissolved inorganic N and P, bridging the gap in the understanding how this important pool of nutrients can promote coral growth and reef development. We report the results of a series of long-term time course experiments in tightly controlled nutrient conditions in the laboratory^43–45^ to test the direct effects of dissolved inorganic N and P on the growth of ten common coral species. Next, we used the results of our physiological and stable isotope labelling experiments in a mathematical model to establish the mechanism by which N and P are transferred from the symbiont to the host. Finally, we used a large-scale natural nutrient-enrichment experiment in combination with stable isotope tracing to demonstrate that these processes can be critical to growth of coral communities in the wild.

## N and P sustaining rapid coral growth

**Fig. 1:**
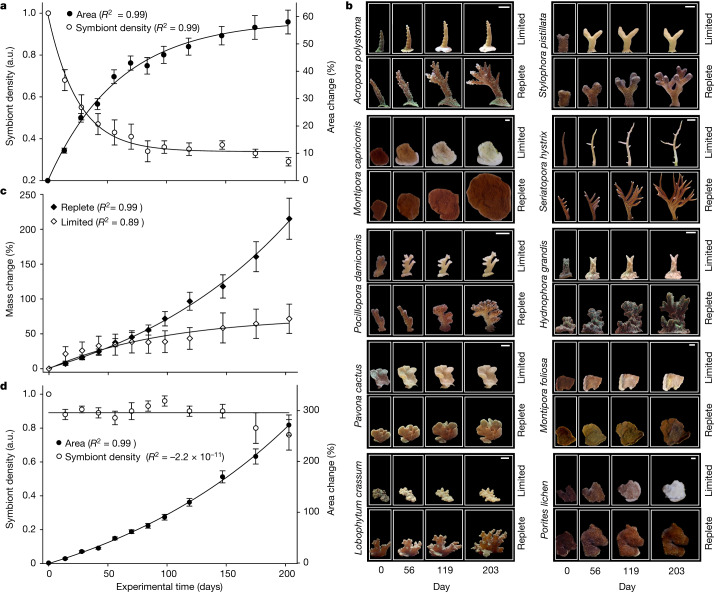
Time course of coral growth and calcification over 203 days in nutrient-replete and nutrient-limited conditions in the absence of particulate food. **a**, Changes in coral surface area and symbiont density in nutrient-limited conditions. a.u., arbitrary units. **b**, Representative replicate samples of each experimental coral species showing visual changes in coral area and symbiont density-dependent colour over time in nutrient-limited (top) and nutrient-replete (bottom) conditions. Scale bars, 1 cm. **c**, Changes in coral mass in nutrient-limited and nutrient-replete conditions. Mass changes of scleractinian coral species are driven mostly by the growth of the calcaraeous skeletons. **d**, Changes in coral surface area and symbiont density in nutrient-replete conditions. Data are presented as mean ± s.e.m. for *n* = 10 different coral species for each of the 2 experimental conditions (6–10 replicate colonies were analysed for each species). Data points are fitted with exponential decrease, increase or saturation functions. *R*^2^ values indicating quality of fits are shown. Horizontal linear fit through sample mean describes symbiont density over time. Source data

We exposed 10 replicate colonies for each of the 9 hard and one soft coral species to either replete or limiting concentrations of nitrate and phosphate for more than 6.5 months (Supplementary Fig. 1 and Methods). Nutrients in these chemical forms cannot be directly assimilated by the animal hosts and particulate material that could potentially serve as food was removed by UV sterilization and microfiltration before the water entered the flow-through tanks with the experimental corals. For corals kept in the nutrient-limited aquarium system, growth and calcification started to stagnate after around 50 days (Fig. 1a–c). Over the same period, these corals lost more than half of their symbiont population, resulting in a bleached appearance (Fig. 1a,b). By contrast, in the nutrient-replete system, the corals grew and calcified at an exponential rate (Fig. 1b–d), whereas symbiont density remained constant (Fig. 1b,d). By the end of the experiment, the coral area covered with live tissue had increased by approximately threefold in corals living in nutrient-replete conditions, indicating a concomitant increase of host and symbiont biomass. We used a subset of these experimental corals (*Montipora foliosa, Montipora capricornis, Acropora polystoma* and *Stylophora pistillata)* to determine the N and P content of the tissue covering the expanded coral area. The increase in host biomass per colony grown under nutrient-replete conditions corresponds to an average gain of 0.25 mg P and 2.44 mg N. Because the coral hosts were deprived of particulate food in our experiments, this gain in N and P indicates an efficient uptake of dissolved inorganic N and P by the symbiont and subsequent transfer to the host.

**Fig. 2:**
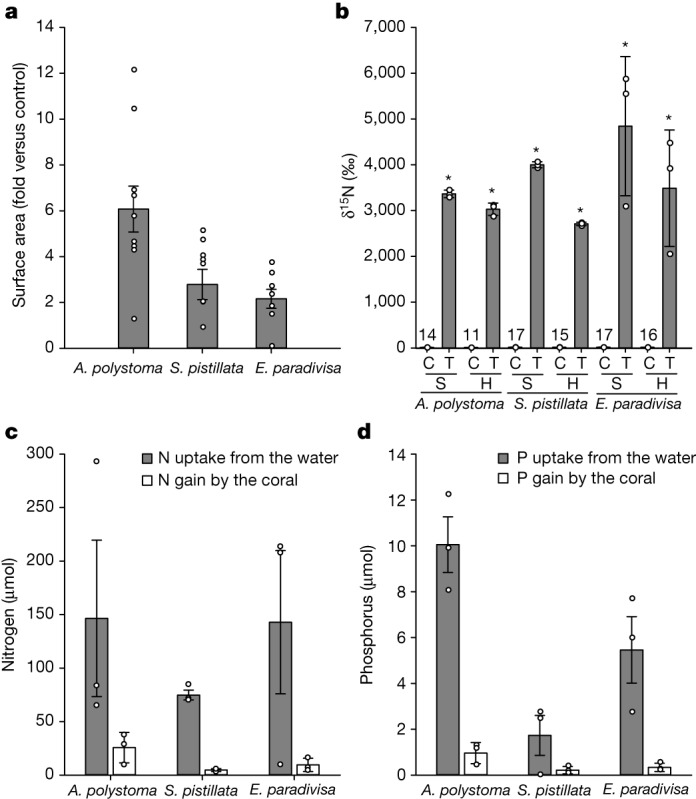
Uptake and assimilation of dissolved inorganic N and P. **a**, Increase in coral surface area in response to defined pulses with ^15^NO_3_ and PO_4_ relative to untreated controls. *n* is the number of independent individuals per species for treatments (T) and controls (C). *A. polystoma*: *n*_t_ = 10, *n*_c_ = 10; *S. pistillata*: *n*_t_ = 9, *n*_c_ = 8; *E. paradivisa*: *n*_t_ = 7, *n*_c_ = 7. **b**, δ^15^N (‰) of host tissue (H) and symbionts (S) in response to treatment with defined ^15^NO_3_ pulses compared with untreated controls. Numbers above control bars indicate the δ^15^N (‰) values. Data are mean ± s.d. of replicate samples for each species (*n* = 3 independent host tissue or symbiont samples from independent individuals for all species, except *n* = 2 for *E. paradivisa* control). Asterisks indicate statistically significant differences between treatment and control (*n* = 3 independent biological species per symbiont community); *t*-test pairwise comparisons, two-tailed *P*_H_ = 0.0002, *P*_S_ = 0.0007. **c**,**d**, The amount of N and P taken up from the water and the N and P gain of host tissue and symbionts over 217 days. Data are mean normalized to the coral surface area. Error bars represent the s.e.m. of N and P uptake from the water from *n* = 3 independent measurements on three different days and of N and P gain by the coral from *n* = 3 independent tissue samples from different individuals. Source data

To test our hypothesis that the N and P responsible for sustaining host growth in our experiments was supplied by the symbionts, we conducted a stable isotope labelling experiment. Three coral species (*Euphyllia paradivisa*, *A. polystoma* and *S. pistillata*) were exposed to daily 2-h pulses of controlled amounts of ^15^N-enriched NO_3_ and PO_4_ for 5 days per week over a period of more than 8 months in separate compartments of the otherwise nutrient-limited experimental system (Supplementary Fig. 1 and Methods). Corals exposed to dissolved inorganic nutrient pulses grew about 3.7-fold more than the controls (Fig. 2a). By the end of our experiments, the ^15^N values (δ^15^N) for both symbionts and host tissue were more than 200-fold higher than those of the controls (Fig. 2b). The significant enrichment of ^15^N in the host tissue provides direct evidence that the gain in host N was achieved through the uptake of dissolved ^15^NO_3_ by the symbiont. Over the duration of the experiment, each colony took up on average around 121 µmol N and 5.7 µmol P from the water in dissolved inorganic form (Fig. 2c,d). This uptake resulted in a gain of around 4.8 µmol N and 0.14 µmol P by the symbiont tissue and around 8.4 µmol N and 0.35 µmol P by the host tissue (Fig. 2c,d). The conversion efficiency of dissolved inorganic nutrients into particulate organic symbiont biomass was around 4.0% for N and 2.5% for P. Remarkably, the conversion efficiency was higher in the host tissue (approximately 7.0% for N and 6.2% for P), indicating that the host represents a sink for the N and P taken up by the symbiont in dissolved inorganic form. These results show that relatively short pulses of 2 h per day to an otherwise nutrient-limited system can be sufficient to promote coral growth (Fig. 2a). This finding can help to explain the higher coral dominance in reef systems experiencing nutrient pulses of similar duration driven by, for example, internal waves^22,46^ (Extended Data Fig. 1).

Our experiments under controlled conditions establish that the assimilation of inorganic nutrients by the symbiont can fully sustain the growth of symbiotic corals. Moreover, our results also demonstrate that both N and—unexpectedly—P are efficiently transferred from the symbionts to the host. These findings cannot be explained by current mechanistic understanding of the partitioning of P within the symbiosis^4^. However, previous work proposed that corals digest part of their symbiont population to control symbiont numbers^47^. This process takes place in the coral mesenteries after phagocytosis of symbiont cells previously released in the gastrodermal cavity via exocytosis^47^. We hypothesized that coral hosts use this strategy to prevent being overgrown by their symbionts as well as to efficiently acquire N and P required for their growth through the digestion of symbionts. If the coral host does not have access to particulate organic food, as in our experiment, a detailed N and P budget of host and symbiont growth will explain the translocation of these nutrients through symbiont digestion to the host.

## Symbiont pruning to extract N and P

**Fig. 3:**
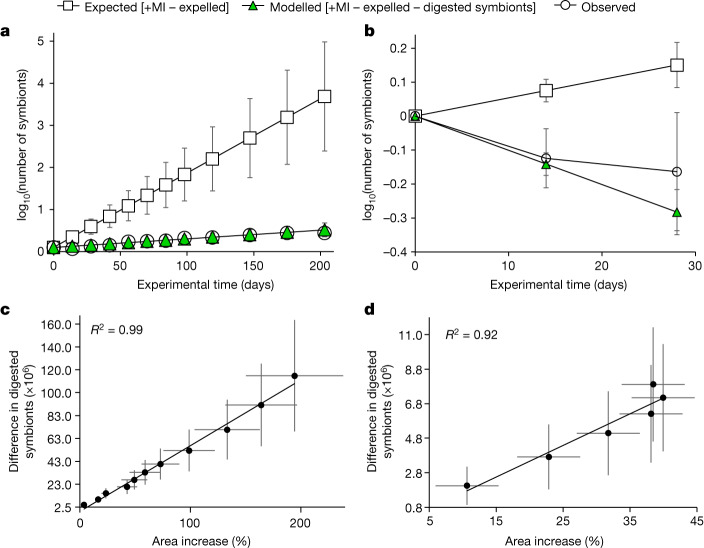
Changes in symbiont numbers in response to the availability of dissolved inorganic nutrients. **a**, Expected expansion of the symbiont population in corals cultured in nutrient-replete conditions based on cell division (mitotic index (+MI)) and expulsion rates over 203 days, the observed increase in symbiont numbers and a model reproducing the observed increases in numbers based on the continuous removal of symbionts by the host. **b**, Expected increase, and observed and modelled decrease in symbiont numbers in corals cultured under nutrient-limited conditions. **c**,**d**, Correlation between the number of missing symbionts and the growth of corals measured as area increase under nutrient-replete conditions over 203 days (**c**) and nutrient-limited conditions over 84 days (**d**). **a**–**d**, Points represent normalized average ± s.e.m. for four coral species (*A. polystoma*, *S. pistillata*, *M. capricornis* and *M. foliosa*). The average for each species represents 6–10 colonies. *R*^2^ values are shown. Correlation between variables of each fit in **c**,**d** are significant (*P* < 0.0001). Source data

Next, we quantified the proportion of the symbiont population digested by the coral host in our experiments (symbiont digestion rate). Based on the individual mitotic indices of symbionts from *M. foliosa, M. capricornis, A. polystoma* and *S. pistillata* cultured in nutrient-replete conditions, we found that the average daily proliferation rate was 4.2 ± 0.8% of the total symbiont population. Then, we used the species-specific mitotic indices to calculate the total number of symbiont cells per coral colony that can be expected from the proliferation of the initial symbiont stock over the duration of the 203-day experiment (Figs. 1 and 3a). This theoretical number exceeded the actual numbers of symbionts per coral colony measured at the end of the experiment by four orders of magnitude, even when the numbers of symbiont cells expelled from the corals was subtracted (average expulsion rate ≈ 0.02 ± 0.01% of the symbiont population per day) (Fig. 3a). The low symbiont expulsion rates determined in our experiment match the previously published range^48^, indicating that a further removal of symbiont cells is indeed happening in these organisms. We calculated that 3.5 ± 0.7% of the symbiont population must be digested by the host per day to hindcast the real increase in symbiont numbers due to the growth-related expansion of the coral area at the observed constant cell densities (Figs. 1 and 3a).

**Extended Data Fig. 2:**
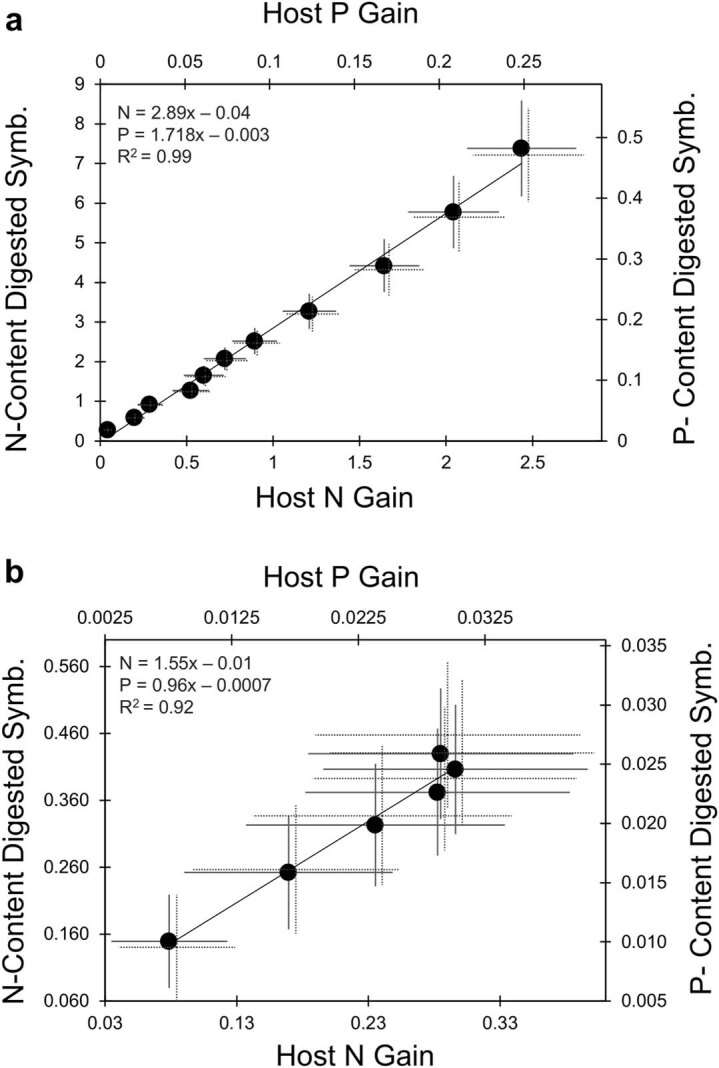
Correlation of the N and P content of the digested symbiont fraction with the N and P content gained by the host during growth over 203 days in the nutrient replete (a) and nutrient limited (b) conditions. Data points represent means ± standard error (solid line error bars: changes in N, dotted line error bars: P). n = 4 independent species: *A. polystoma, S. pistillata, M. capricornis, M. foliosa*. 6–10 colonies were analysed per species. Equations of linear fits and R^2^ are given in the chart. Correlations between variables of each fit are significant with p < 0.0001.

We then determined whether the digestion of symbionts provides enough N and P to sustain coral growth. We used the 3.5 ± 0.7% symbiont digestion rate to calculate the amount of N and P that is contained in the fraction of symbionts that makes up the difference between the expected and the measured symbiont numbers at the end of the experiment. The N and P amounts related to this unaccounted symbiont fraction correspond to around 3.0 times and 1.9 times the amounts of N and P, respectively, that were gained by the host (Extended Data Fig. 2a), sufficient to explain the growth measured in the experiment. Furthermore, the number of unaccounted symbionts shows a significant positive linear relationship with the measured increase in coral area at the corresponding experimental timepoints (Fig. 3c). This correlation clearly indicates that the coral host ‘farms’ the symbionts and regularly digests a proportion of their population to fulfil its nutritional demand. This is further supported by the fact that after transfer to nutrient limiting conditions, corals continued to grow for around 4 weeks at similar rates to their counterparts in the nutrient-replete system (Fig. 1a,c). Their associated areal gain was strongly correlated with the concomitant decrease in symbiont numbers (Fig. 1a). Using the previously determined daily symbiont digestion rate of 3.5 ± 0.7% and an average daily cell division rate of 0.84 ± 0.16% calculated for corals cultured in nutrient-limited conditions, our model output clearly matches the observed symbiont numbers. Thus, we provide an independent experimental proof for the accuracy of the model (Fig. 3b). Moreover, the calculated number of digested symbionts under nutrient-limited conditions shows again a positive linear relationship with the measured increase in coral area during the early experimental timepoints (Fig. 3d). Also, the N and P content of the missing symbionts can provide sufficient N and P to fuel the growth of the host over these first four weeks (Extended Data Fig. 2b). Notably, the daily symbiont digestion rate of 3.5 ± 0.7% determined from our experiments is in excellent agreement with the average frequency of degraded symbiont cells (3.8%) in the tissue of 8 coral species collected from Okinawa reefs^47^.

## Explaining the N budget of corals

We explored whether the mechanism of dissolved inorganic nitrogen uptake and translocation through symbiont digestion established in our laboratory experiments can help to explain the N budget of symbiotic corals in natural reef environments at the ecosystem level. To this end, we studied a unique large-scale natural nutrient-enrichment experiment in the Chagos Archipelago. This complex of mostly uninhabited reef atolls in the central equatorial Indian Ocean consists of islands where dense seabird populations introduce large amounts of guano to the surrounding waters, along with islands where seabird densities and guano input are low^38^. The guano production by seabird colonies results in an increase of nitrate and phosphate concentrations in adjacent reef waters^28,38,39,49^ (Extended Data Fig. 1 and Extended Data Table 1). Near coral islands with high seabird densities, elevated PO_4_ concentrations of 30 nM have been recorded, compared with 5 nM near islands with low seabird densities^39^. Close to the shore of seabird islands, NO_3_ concentrations can reach values as high as 10–20 μM, whereas measurements at reference sites with low seabird densities^28,49^ can return values in the range between 0.01 and 0.73 μM.

**Fig. 4:**
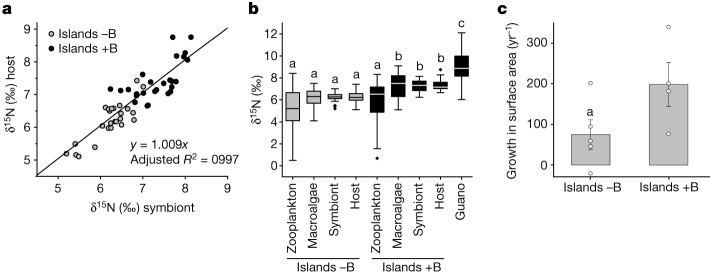
Assimilation of ^15^N-enriched nitrogen by *Acropora* sp. in reefs adjacent to islands with dense seabird populations in the Chagos Archipelago compared to islands with low numbers of seabirds. **a**, δ^15^N for host tissue and associated symbionts from islands with high (+B) or low (−B) densities of seabirds. The equation of the linear fit and adjusted *R*^2^ are shown. **b**, δ^15^N of biologically independent samples from islands with high or low densities of seabirds for coral host (*n*_+B_ = 27, *n*_−B_ = 25), symbionts (*n*_+B_ = 27, *n*_−B_ = 25), their nitrogen sources (zooplankton (*n*_+B_ = 18, *n*_−B_ = 17) and bird guano (*n* = 22)). Macroalgae (*n*_+B_ = 55, *n*_−B_ = 45) from the same reefs are included for comparison. In box plots, the centre line shows the median, the box encompasses 25th and 75th percentiles, and whiskers extend to minimum and maximum values of the dataset. The filled circles display outliers. Significant differences (indicated by different letters) between samples were determined by one-way ANOVA (*P* < 0.001) followed by pairwise multiple comparison (Holm–Sidak method, *P *< 0.05). The number of biologically independent samples (in the range of *n* = 17–55) are provided in Methods. **c**, Growth of *Acropora* sp. colonies measured as expansion of surface area per year. Data are mean ± s.e.m., a indicates a significant difference between the datasets (*t*-test pairwise comparison, two-tailed *P* = 0.04). Samples represent independent islands (*n*_+B_ = 4; *n*_–B_ = 5) across three atolls in the Chagos Archipelago. Source data

Because the δ^15^N of dissolved inorganic N derived from guano is high compared with normal reef levels^28,38^, it can be used as a natural tracer for the uptake of dissolved inorganic N and subsequent partitioning within the symbiotic association^49^. As the assimilation of nitrate is restricted to the symbiont, any increase in host δ^15^N can be unequivocally assigned to the dissolved N uptake by the symbiont and subsequent translocation to the host^18^. Likewise, NH_4_^+^, if present in the water, will be incorporated mostly by the symbiont^16^. Therefore, it can be confidently assumed that a large amount of dissolved inorganic nitrogen will enter the symbiotic association via assimilation by the symbionts. Comparing the δ^15^N values of host tissue and symbionts of *Acropora* corals, we find that the presence of seabirds results in a significantly increased isotopic values of both host and symbiont (Fig. 4a,b). The average δ^15^N of the host coral tissue on reefs next to islands with seabirds is comparable to the values obtained from macroalgae in the same reef habitats^38^, apparently assigning the corals to the same trophic level as a fully N- and P-autotrophic organism (Fig. 4b). By contrast, zooplankton collected from reefs of islands with seabirds did not exhibit significantly higher δ^15^N values compared with samples from islands without seabirds, indicating a dominantly allochthonous origin of this organic N supply for the corals.

Applying a geochemical source mixing model^49^ and using the δ^15^N values of guano and zooplankton as endmembers, we find that around 50% of coral host N can be traced back to guano-derived N and thus to the primary uptake by the symbionts. Consequently, N acquisition by heterotrophic feeding on zooplankton contributes less than 50% to the N budget of the coral host. Across a multi-year in situ tagging experiment, the area of *Acropora* colonies showed growth rates about two times higher in waters surrounding islands with high seabird densities compared with the reefs around islands without large seabird colonies (Fig. 4c). These data are consistent with the approximately threefold increase in linear extension of *Acropora* after transplantation to NO_3_-enriched reef water close to seabird colonies^28^, revealing the direct benefits seabird-mediated nutrient enrichment to coral growth at the ecosystem scale. In the absence of alternative known major translocation mechanisms, the symbiont digestion pathway established by our laboratory experiments offers a plausible explanation for the significant accumulation of seabird-derived nitrogen by the coral host, indicative of a major contribution symbiont digestion to the nitrogen budget of coral animals in natural reefs.

## Benefits of photosynthetic symbionts

**Fig. 5:**
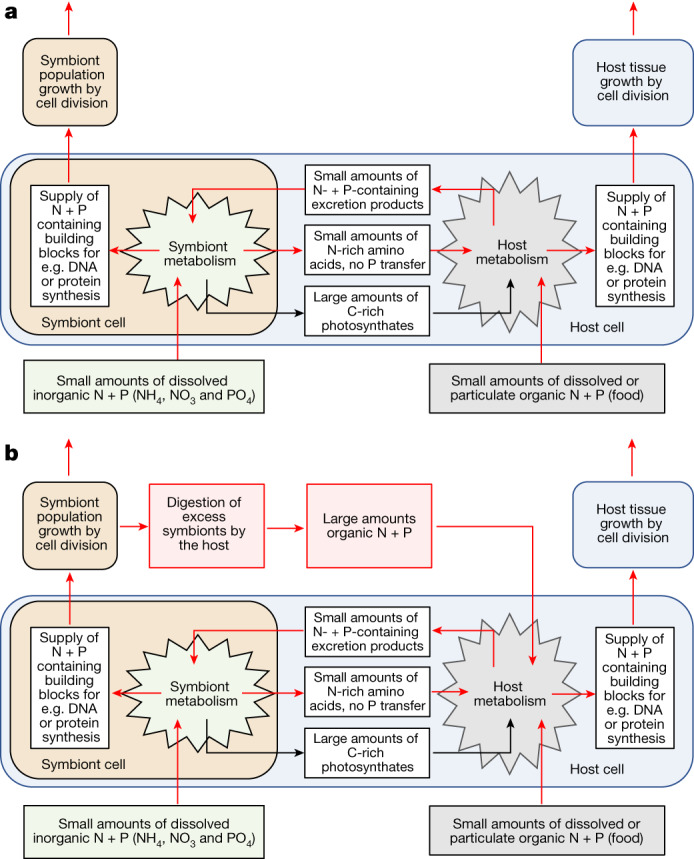
Schematic depiction of N and P uptake, partitioning and recycling mechanisms within the coral–dinoflagellate symbiosis. **a**, In the traditional view, large amounts of C along with modest amounts of N and essentially no P are released from the symbionts. Accordingly, the uptake and assimilation of dissolved inorganic N and P by the symbiont provides limited benefits to the host. The host relies largely on heterotrophic uptake of the essential N and P to obtain the building blocks for its growth. **b**, Based on our findings, the host can gain full access to the pool of dissolved inorganic nutrients, that would otherwise not be accessible to coral animals, by acquiring N and P through feeding on symbionts. If sufficiently large amounts of dissolved inorganic N and P are available to the symbiont, the host can sustain its growth and metabolic demands exclusively through symbiont farming and digestion. In nutrient-limited conditions, the symbiotic association can exploit both major pools of nutrients, dissolved inorganic forms of N and P as well as dissolved and particulate organic forms of N and P. In well-lit, clear, warm and nutrient-poor waters, the ability of the coral to reciprocally transfer these vital N and P compounds between the partners of the symbiotic association gains them an evolutionary and ecological advantage over plants or animals that are limited to accessing one or the other nutrient pool.

Our experimental findings show that coral animals can farm their symbionts and feed on the symbiont stock to access a pool of dissolved inorganic N and P in the surrounding waters that would not otherwise be accessible to them (Fig. 5). If enough dissolved N and P is available, feeding on symbionts represents a mechanism to fully meet the nutrient demands of coral growth. Under these conditions, the corals function in essence similar to fully C-, N- or P-autotrophic organisms such as plants, algae and photosynthetic prokaryotes. When food and nutrient supplies are too low, the continued consumption of symbionts at a constant digestion rate can act as an emergency measure to maintain their productivity for a limited period until the symbiont stock is depleted and the corals bleach (Figs. 1 and 3). However, if the corals cannot take in N and P in quantities that meet the demand of both partners of the symbiosis^50^, feeding on their symbionts in nutrient-depleted waters can eventually lead to coral death.

The gains for the coral host from feeding on their symbionts come in addition to the well-known benefits provided by their symbionts, such as transfer of C-rich photosynthates to the host and retention and recycling of its N- and P-rich excretion products^11,14^. Furthermore, feeding on the symbionts ideally complements heterotrophic N and P acquisition of the host because it taps into a different nutrient pool (Extended Data Fig. 1 and Extended Data Tables 1 and 2). Of note, growth of *Acropora* sp. is enhanced by a phytoplankton-dominated diet, indicating that their digestive machinery is indeed well-suited to process algal cells^51^.

## Corals feed on symbionts to succeed

Our results demonstrate that mixotrophic symbiotic coral animals can satisfy a substantial amount of their N and P demand through the assimilation of dissolved inorganic nutrients by the symbionts and subsequent translocation to the host through feeding on the symbionts. Thus we demonstrate that both the symbionts and the host gain growth-related benefits through the efficient, reciprocal exchange of the essential cellular nutrients N and P (Fig. 5). As a symbiotic association, corals can fully exploit both the pool of organic N and P through host heterotrophy and the pool of dissolved inorganic N and P through autotrophy of the symbiont. Since both organic and inorganic sources of N and P are overall scarce in oligotrophic tropical waters^10^, the symbiotic lifestyle offers corals a truly competitive edge over exclusively heterotrophic animals that rely solely on N and P in organic forms, or exclusively autotrophic plants such as macroalgae that are restricted to N and P in dissolved inorganic form. The capability of the symbiotic partners to benefit together from N and P sources that would not be accessible to either the coral animal or the photosynthetic dinoflagellates outside of their association enables them to thrive in environments where only one of the nutrient sources would not be sufficient to sustain growth. The fully closed cycle of reciprocal N and P exchange between the symbiotic partners (Fig. 5) can explain the evolutionary and ecological success of symbiotic corals in well-lit, nutrient-limited warm water habitats. In sum, our study provides a missing piece of the puzzle required to explain the success of coral reefs in seemingly barren ocean waters that has intrigued scientists since Darwin’s pioneering work to explain why coral reefs grow where they do^1^.

## Implications for coral reef survival

Anthropogenic nutrient enrichment is a well-documented threat to coral reef survival that will become more serious as coastal populations increase in the future^52–55^. Conversely, global warming is predicted to drive regional depletion of dissolved inorganic nutrients in surface waters by increasing thermal stratification of the oceans and deepening of the nutricline, especially near some of the coral reef islands and atolls that span the oligotrophic tropical oceans^31^. Our findings suggest that this disturbance of the nutrient environment may increase stress levels on affected coral reefs. The lack of supply with N and P in dissolved inorganic form will directly deprive symbiotic corals of a major nutrient source, and will also have negative effects on their other major source of organic N and P through the overall reduced productivity of nutrient-depleted waters^20^. The lack of sufficient provision with nutrients has the potential to induce coral bleaching and death in addition to the heat stress-induced loss of symbionts^52^, aggravating the severe effects of global warming on the livelihoods of hundreds of millions of people and a large part of marine biodiversity that depends on functioning coral reefs^56^. Nutrient acquisition via digestion of symbionts by the coral host will probably have an important role in determining the response of coral reefs to future change.

## Methods

An overview of the methods (M1–M21) applied in the context of the experiments is provided in Supplementary Fig. 1.

### Coral culture

The experimental corals (Supplementary Table 1) have been cultured and propagated by fragmentation in the experimental coral mesocosm facility of the Coral Reef Laboratory at the University of Southampton since 2008 (refs. ^43,57^). The coral models for the present study were selected as they have been used previously in physiological experiments by others. Moreover, they represent common species with a wide geographic distribution. Additionally, they include different colony morphologies to demonstrate the broad applicability of our findings. *Acropora* and *Stylophora* were selected as they feature in many previous studies on nutrient effects on symbiotic corals and they are also widely distributed reef-building corals. *E. paradivisa* with its phaceloid growth and large polyp size was included to demonstrate that the effects are reproducible independent from the morphological features of the experimental model.

Temperatures (~27 °C) and salinity (~33 practical salinity units (psu)) in the experimental aquaria were monitored regularly and kept constant during long-term culture and experimentation. Aquaria were illuminated by metal halide lamps (Aqualine 10,000, Aqua Medic), operated on a 12-h light/dark cycle, exposing the corals to a light intensity of ~105 µmol m^−2^ s^−1^. Turbulent flow was generated using a Turbelle Nanostream 6045 (Tunze), operated at a flow rate of 4,500 l h^−1^. Corals were kept in nutrient-replete conditions ([NO_3_] ≈ 12 µM, [PO_4_] ≈ 3 µM), simulating nutrient environments that have been previously described for reefs with increased coral growth rates (Extended Data Fig. 1 and Extended Data Table 1). To test the response of different corals to nutrient-replete and nutrient-limited conditions, ten individual microcolonies were produced from fragments of one mother colony for each of the 10 experimental species (10 species × 10 colonies = 100 colonies). Coral fragments were attached to ceramic tiles using two-component epoxy resin (D-D Aquascape; D-D The Aquarium Solution) and allowed to regenerate for >4 weeks prior to the start of the experiments. Five colonies per species were used in nutrient-replete and nutrient-limited conditions, respectively. For the nutrient pulse (^15^N-labelling) experiment, 7–10 replicate colonies per species were used for treatment and control conditions, respectively. Unless indicated otherwise, the taxonomic background of the symbionts associated with experimental corals (Supplementary Table 1) was confirmed by the time of the experiments through PCR-amplification and sequencing of the internal transcribed spacer 2 (ITS2) regions of the nuclear ribosomal RNA genes as described^58,59^, using the classification of symbiont species from ref. ^60^ (Supplementary Fig. 1, M1).

### Maintenance of nutrient environments for long-term experiments

Experimental corals were kept in separate flow-through tanks under nutrient-replete conditions, as described above, and in a nutrient-limited system ([NO_3_] ≈ 0.7 µM, [PO_4_] ≈ 0.13 µM) with the same layout. Temperature, salinity, light and flow conditions in the nutrient-limited system were identical compared to the nutrient-replete system. Nitrate was removed continuously from the system using a Nitrate reactor (Aqua Medic), whereas phosphate was removed by filtering the water through a RowaPhos Matrix (D-D The Aquarium Solution). Ammonium levels in both, the nutrient-replete and limited systems, were constantly low^45^. Particles that could potentially serve as coral food were removed from the seawater by microfiltration with a 10-in. FilterPlus Housing fitted with a 1-µm polypropylene (PP) filter (TradeMark Aquatics) and further sterilized by UV treatment with a V2 Vecton 120 UV Steriliser (Tropical Marine Centre) before the water entered the compartments with the experimental corals. The experimental tanks and the ceramic tiles were regularly cleaned to remove any algal films (Supplementary Fig. 1, M2).

### Nutrient pulse treatments and ^15^N labelling

Experimental corals attached to ceramic tiles were kept in 3.5-l food grade polyethylene tanks that were submerged in a large holding tank connected to the nutrient-limited system. A laminar water flow over the top of the tank was generated by current pumps and resulted in turbulent water movement inside the submerged experimental tanks. Five days per week, 1 h after the start of the light period, these tanks were raised 5 cm above surface for 2 h so that no water exchange with the rest of the system occurred. Water flow and aeration inside the isolated treatment tanks were maintained with air stones. Elevating the corals, together with the tanks, minimized the disturbance of the corals prior to the treatment and all polyps remained expanded. Once the tanks were isolated, the water in each treatment tank was spiked with 1 ml of ^15^N-enriched (10%) NO_3_ and PO_4_ stock solutions, to reach an average concentration of ~10 µm NO_3_ and ~3 µM PO_4_. A second spike was delivered after 1 h. The control tank was dosed with 2 ml of Milli-Q water. The tanks were then covered with a lid to prevent aerosols and micro-droplets generated by the air bubbles to enter the main system. By the end of the 2-h spiking treatment, the tanks were removed from the system and the corals were lifted from the treatment tank and washed by dipping in a tank with system water to remove residual nutrient enriched water. Subsequently, the corals were transferred to identical, clean tanks pre-filled with system water that were again submerged in the holding tank. All used tanks were cleaned with demineralized water, dried and stored for the next round of treatments. Great care was taken to avoid contaminations with ^15^N-enriched NO_3_. The tiles holding the corals were cleaned once per week with a toothbrush to prevent algal growth. The experiment was conducted for 217 days before the samples were collected and frozen for downstream analyses (Supplementary Fig. 1, M3).

### Nutrient uptake rates

On days 1, 3, and 5 of the final week of the nutrient pulse (^15^N-labelling) experiment, nutrient uptake rates were determined. Treated and control corals were isolated from the experimental system in their incubation tanks in a defined volume of nutrient-limited water. N and P concentrations in the incubation tanks were measured in defined intervals (5, 30, 60, 65, 90 and 120 min) over the 2-h incubation period after administering the nutrient pulse to the treatment tanks. For each timepoint, water samples (50 ml) from each of the isolated treatment and control tanks containing were collected. The samples were filtered through a 0.2-µm Minisart Syringe Filter into a sterile 50 ml tube (Falcon 50 ml Conical Centrifuge Tubes, Fisher Scientific) and immediately frozen for later analysis by an AutoAnalyzer (QuAAtro39 segmented flow auto-analyser, Seal Analytical).

To determine the amount of nutrients taken up during the incubation period by the control and treatment corals, the amount of nutrients removed from the water was integrated. The values measured for corals kept at nutrient-limited control conditions were subtracted as background from the corresponding nutrient removal values in the nutrient-spiked tanks. Average values were calculated for the corresponding timepoints of the measurements on the three different days. The resulting values were set equal to the amounts of nutrients taken up by the corals and normalized to the area of the corals. To determine the N and P amounts take up over the duration of the experiment, the uptake rates over time were corrected for the change in surface area of the replicate colonies (Supplementary Fig. 1, M4).

### Monitoring the effects of different nutrient environments

#### Standardized imaging

Corals were photographed with a digital Olympus Tough F2.0 camera at the start of the experiments and then at regular 2- to 3-week intervals, to measure growth and symbiont density in response to the nutrient treatments. A standardized imaging procedure was followed: first the coral colonies were transferred to a glass tank pre-filled with water from their specific treatment system (nutrient-limited or replete). The tank was afterwards placed in a defined position inside a purpose-built black cabinet lit with white LED lights fitted with diffuser screens fixed to both sides and to the top lid of the cabinet, ensuring even and reproducible illumination across samples. Photographs were subsequently taken with room lights switched off to avoid changes in the light exposure. Camera settings were kept the same for all images. Colonies were photographed always from the same orientation and against size and colour scales for reference (Coral Health Chart, CoralWatch). Branching colonies were photographed from the side and foliose or encrusting colonies from the top (Supplementary Fig. 1, M5).

#### Coral growth by surface area

The visible surface area of each coral colony was determined from the standardized photographs using the polygon tool in FIJI/ImageJ (version 1.53). The total surface area of each specimen for a defined set of species, was measured after removal of the organic fraction. Due to the specific challenges imposed by the different colony morphologies, the total area of branching corals was determined using the paraffin wax dipping technique^61^ and the foil wrapping method for plate-forming shaped corals. We confirmed that the two protocols yielded comparable results for our sample types using a subset of representative samples of each species. Therefore, the minimal deviations that may result from the use of different methods between species with different growth morphologies would become part of the intraspecific variability and part of the error estimate. For foil wrapping, coral skeletons were covered with aluminium foil which was cut and molded to fit the shape of the colony. The aluminium foil was then trimmed around the edges to match the skeleton boundaries and weighed. To convert foil weight into surface area the coral, the conversion factor was obtained by weighing a series of pieces of aluminium foil with a known surface area. To determine the surface area using the wax dipping technique, coral skeletons were dipped into a 1-l glass beaker containing melted paraffin wax (60 °C) for 3 s and carefully shaken to remove excess wax. After 5 min, corals were weighed, dipped again into wax for 3 s, air dried for 5 min and re-weighed. The weight difference between the first and second coating was used to determine the weight increment caused by the second coating. To determine the surface skeletal area, the following regression was used^4^: surface area (cm^2^) = 34.32 (cm^2^ g^−1^) × mass (g). The total area values were used to extrapolate the intermediate values of growth over time by scaling the real final area values to the changes in visible surface area determined from the standardized photographs. This data transformation is permitted since the changes in visible surface area were proportional to the changes in weight, with the latter being proportional to the 3D area under constant growth conditions (Supplementary Fig. 1, M6).

#### Coral growth by mass

Identical tiles from the same batch were used to mount all replicate colonies. Additionally, a set of three tiles were kept empty and incubated next to the coral colonies in each experimental system. All tiles were regularly cleaned along with the tiles holding the corals. Corals on their attachment tiles were placed on adsorbent paper for a 10-s drip-off period to remove residual water. Mass of the coral colonies was then determined using a Fisherbrand analytical balance. The reference tiles were weighed at each timepoint of data collection and their average value was subtracted from the mass of the coral colonies at the respective timepoints. Mass readings were taken on the same days as the photographs (Supplementary Fig. 1, M7).

#### Processing of coral samples for downstream experiments

At the end of the experiments, corals were removed from the experimental system and placed on absorbent paper for a 10 s drip-off period. Subsequently, the samples were frozen individually at −20 °C for downstream analysis. To obtain host and symbionts fractions, coral tissue was quantitatively removed with an airbrush with Milli-Q water, homogenized and the symbiont fraction was separated by centrifugation at 1,500*g* (10 min, 4 °C). The host tissue contained in the supernatant was re-centrifuged at 16,000*g* (10 min, 4 °C) and kept at –20 °C for further analysis. Symbionts pellets were washed and re-centrifuged at 16,000*g* (10 min, 4 °C), and divided in two aliquots. For mitotic index determination, pellets were fixed with 10% formaldehyde and suspended in sterile artificial seawater at 4 °C overnight. For isotope analysis pellets were frozen at −20 °C (Supplementary Fig. 1, M8).

### Symbionts analysis

#### Symbiont densities and actual number of symbionts per colony

We established a procedure to deduce the symbiont densities from the mean grey value (MGV) of the coral photographs. The MGV, the sum of the grey-scaled values of all the pixels in a selected area of an image divided by the number of pixels, depends to a large extend on the amount of photosynthetic pigments of the symbiont shining through the host tissue. Under constant environmental conditions, the pigment density per symbiont cell does not change and, accordingly, the “darkness” of the host tissue is proportional to the symbiont density^62^. The MGV was determined for the whole visible area of every coral using the polygon tool in FIJI/ImageJ (version 1.53). To extrapolate symbiont densities from MGV values, a calibration curve was constructed using four different coral species (*M. foliosa*, *M. capricornis*, *A. polystoma* and *S. pistillata*). Replicate colonies of each species acclimated to nutrient-replete conditions were transferred to the nutrient-limited system. Replicate samples were removed for each species and photographed for MGV determination at day 0 (transfer day) and at three regular timepoints spread over the 70-day experiment. The coral surface area was determined by the aluminium foil or wax dipping techniques as described above and the symbionts were isolated following established protocols (see below) and quantified by flow cytometry analysis using a CytoSense flow cytometer (CytoBuoy) with the CytoUSB v5.7.5.7 data acquisition software. Symbiont densities calculated from these data were plotted against MGV values to obtain the calibration curve (*y* = 2*E* + 6 × e^−0.011X^, *r*^2^ = 0.70). Subsequently, symbiont densities at the different experimental timepoints were deduced from the MGV values of the standardized coral images. The actual total number of symbionts per colony on a given day (*S*_*n* exp._) was calculated from the experimentally determined symbiont density and the area at the given timepoint (Supplementary Fig. 1, M9).

#### Mitotic indices

Symbiont cells fixed in 10% formaldehyde were analysed using CytoBuoy CytoSense flow cytometer and CytoUSB v5.7.5.7 data acquisition software. All samples were run at a flow rate of ~5 μl s^−1^ and photomultiplier tube voltage 100 mv. Cells were excited at 488 nm and detected using a 588 nm band-pass filter (red fluorescence) trigger (100 mV). Symbiont cells were screened using forward scatter and side scatter to remove debris. Symbiodiniaceae cells are strongly autofluorescent, thus, to differentiate between singles cells and cells undergoing cytokinesis, they were analysed using forward scatter against a red (chlorophyll) fluorescence (total fl-red). The mitotic index was determined as the ratio between the number of a population’s cells undergoing cytokinesis to its total number of cells (Supplementary Fig. 1, M10).

#### Symbiont expulsion rate

The expulsion of symbionts from *S. pistillata*, *A. polystoma* and *M. foliosa* under nutrient-replete conditions was measured by collecting all the symbionts released in the water during a 24 h period. Three colonies per species were placed individually in small replicate tanks (~1 l) and exposed to equal light conditions. Water flow and aeration inside the isolated tanks were maintained using air stones. At the end of 24-h period, the corals were gently swirled in the water to detach any symbionts that may still be adhering to its outer surface. After corals had been removed from the tanks, the complete water volume in each tank was collected in bottles and centrifuged for 10 min at ~1,500*g* to collect the released symbionts. The cell pellet was resuspended in a small volume of water and fixed with 10% formaldehyde. Cell numbers were quantified using a CytoBuoy CytoSense flow cytometer as described above. The expulsion rate (*E*′) was calculated as the percentage of expelled cells in relation to the total number of symbionts of the corresponding coral sample (Supplementary Fig. 1, M11).

#### Symbiont digestion rates

The calculation of the number of digested symbionts assumes that the actual total number of symbionts per colony on a given day (*S*_*n*_) equals the number of symbionts resulting from the proliferation of previous’ days symbiont stock (*P*) minus the number of symbionts expelled per day (*E*) minus the number of digested symbionts (*D*).

$${S}_{n}=P-E-D$$

In this context, *P* is calculated by multiplying the previous day’s symbiont stock with the experimentally determined proliferation rate, the mitotic index (MI, here: 4.2 ± 0.77% per day) and adding this number to the previous day’s symbiont stock:

$$P={S}_{n-1}\times {\rm{MI}}(0.042)+{S}_{n-1}$$

The number of expelled symbionts *E* is calculated by multiplying the previous day’s symbiont stock with the experimentally determined expulsion rate *E*′ (here: 0.02 ± 0.01% per day).

$$E={S}_{n-1}\times {E}^{{\prime} }(0.0002)$$

Consequently, *D* can be determined by subtracting the number of symbionts expelled per day (*E*) and the actual total number of symbionts per colony on a given day (*S*_*n*_) from the number of symbionts resulting from the proliferation of previous’ days symbiont stock (*P*).

$$D=P-E-{S}_{n}$$

For *D* to be valid, *S*_*n*_ needs to match the actual total number of symbionts on the corresponding day deduced from experimental data (*S*_*n* exp._).

Based on the above equations, we calculated the theoretical growth of the initial symbiont stock (*P*) that can be expected from the proliferation rate of 4.2 ± 0.77% per day over the duration of the 203-day experiment. This was contrasted with the actual increase in symbiont numbers due to the growth-related expansion of the coral area (*S*_*n* exp._) at the observed constant symbiont cell densities determined in the experiment. *D* (3.5 ± 0.7% per day) was modelled to create a best-fitting curve in which *P*′ values match the *S*_*n* exp._ values at all timepoints of the experiment.

To model the decline of symbiont numbers in our model corals (*M. foliosa, M. capricornis, S. pistillata* and *A. polystoma*) grown under nutrient-limited conditions, we used the same formula with the division rate of 3.5 ± 0.7% and a lower proliferation rate adjusted for reduced mitotic indices of the symbionts exposed to nutrient-limited conditions (0.8 ± 0.16%) (Supplementary Fig. 1, M12).

### Field sampling

#### Coral sampling

In May 2018, branching *Acropora* sp. colonies were sampled from 9 uninhabited islands in the northern atolls of the Chagos Archipelago, Indian Ocean. Four of the islands had diverse and abundant seabird populations which provide substantial nutrient subsidies to the nearshore marine environment, while five of the islands had few seabirds due to the presence of introduced rats^38,63^. The islands were distributed across three atolls, with high-seabird and low-seabird islands within each atoll (Great Chagos Bank: one island per treatment, Peros Banhos: two islands per treatment, Salomon: one high-seabird island and two low-seabird islands). Across this archipelago, the nitrogen input by seabirds per hectare of island is ~250 times greater on islands with dense seabird populations than on islands with low seabird numbers^38^. Further, all corals used in this study were from the reef flats within the reef crest, within 300 m of shore. The influence of oceanic nutrients is expected to be small and consistent among sites. Likewise, there are no anthropogenic nutrient sources within the study region, which is uninhabited and part of a large marine protected area^38^. All study sites were separated by at least 3 km. Coral colonies of similar size and growth form were haphazardly selected on lagoonal sides of the islands within the atolls, rather than on the ocean facing sides (*n* = 4–10 colonies per island). From each colony, a small fragment (~5 cm) was removed using a chisel. All samples were wrapped in aluminium foil and immediately frozen at −20 °C for later separation of host and symbiont tissue (Supplementary Fig. 1, M13).

#### Monitoring of colony growth

Small branching *Acropora* sp. colonies were tagged using numbered cattle tags tied to nearby substrate. Between 5–7 colonies per site were tagged on 5 islands with high and 4 islands with low seabird densities. All colonies were located within 300 m of shore on the lagoon sides of islands. We re-visited all sites in 2019 and 2021, with 5 sites also re-visited in 2020 yielding data of 9 islands for growth analysis. The change in planar area was used as metric of coral growth, a commonly used non-destructive method that is tightly related to changes in three-dimensional surface area and volume^64,65^. Each tagged colony was photographed from above using a Canon S110 camera with a scale bar placed level with the upper surface of the coral. Planar area was measured in each image by outlining the outer edge of the colony using the polygon tool in FIJI/ImageJ. Change in planar area (cm^2^ day^−1^) was calculated for each colony as the difference between the new surface area and the previous surface area divided by the number of days between measurements (Supplementary Fig. 1, M14).

#### Plankton collection

Zooplankton was sampled in March 2019 and 2021 during the day by towing a plankton net and at night using light traps. For daytime sampling a plankton net with 250 µm mesh size was towed behind a small boat next to sites where corals were collected. At each site, 2–3 tows were conducted along the same area parallel to shore for ~20–30 min. For night sampling 2–3 plankton traps made from wide-mouthed plastic bottles with a funnel opening on one end were set before dusk and picked up the following morning shortly after dawn. Two green glow sticks were placed in each trap as an attractant, and traps were secured to the reef near coral collection sites using cable ties. Immediately upon collection, the contents of the trap were filtered through 250-µm mesh. Both day and night plankton were subdivided. Samples were freeze-dried prior to analysis of the isotopic content (Supplementary Fig. 1, M15).

#### Guano collection

Dried guano was collected in May 2018 from leaves of coastal plants containing red-footed booby (*Sula sula*) nests. Samples were taken from 3 different rat-free islands (*n* = 9–10 per island), with only one sample taken from each plant. Guano was immediately dried at 60 °C for 24–48 h and aliquoted for analysis of the isotopic content (Supplementary Fig. 1, M16).

#### N and P elemental analysis and bulk organic δ^15^N content sample processing

Frozen (−20 °C) symbiont pellets and host homogenate were dried using a Mechatech LyoDry Compact freeze dryer (Mechatech Systems) and homogenized to a fine powder with a mortar and pestle for elemental analysis (N and P) and bulk organic δ^15^N content in natural abundance and ^15^N-enriched samples (Supplementary Fig. 1, M17).

#### Bulk organic δ^15^N analysis of natural abundance samples

A Elementar Vario PYRO Cube Elemental Analyser running in CNS mode equipped with a TCD (thermal conductivity detector) interfaced with an Isoprime VisION continuous flow isotope ratio mass spectrometer (IRMS) was used for the analysis. The samples were weighed out in clean tin capsules on a Sartorius ME5 micro balance and were then combusted at 1,120 °C with the addition of pure oxygen. The resulting combustion gases of NO_x_ were subsequently reduced to N_2_ in the reduction column which was held at 850 °C. The elemental ratios were determined by the TCD, the isotope ratios by the IRMS. Sulfanilamide was used as an elemental standard for %N. For the normalization of the isotope ratios, we used USGS 40 and USGS 41 as international reference materials (United States Geological Survey). We furthermore used suitable quality control materials such as the internal High Organic Content Sediment Standard (HOCS, Elemental Microanalysis), which was used to calculate the instrument precision (Supplementary Fig. 1, M18).

#### Bulk organic analysis of δ^15^N-enriched samples

For this the Elementar Vario Isotope Select Elemental Analyser was run in CN mode and interfaced with an Isoprime 100 continuous flow IRMS. The samples were weighed out in clean tin capsules on a Sartorius MP3 micro balance and were then combusted at 950 °C with the addition of pure oxygen. The resulting combustion gases of NO_x_ were subsequently reduced to N_2_ in the reduction column which was held at 550 °C. The elemental ratios were determined by the TCD, the isotope ratios by the IRMS.

We used acetanilide as an elemental standard for %C and %N. For the normalization of the isotope ratios, we used USGS 40 and USGS 41a as international reference materials (United States Geological Survey). We furthermore used suitable quality control materials such as the Protein Standard (Elemental Microanalysis), which was used to calculate the precision of the run as well as any memory effects. δ^15^N values (‰) were calculated as follows: δ^15^N = [(*R*_sample_/*R*_standard_) − 1] × 1,000] where R is the ratio of the heavy isotope (^15^N) to the light isotope (^14^N) of the sample or standard (Supplementary Fig. 1, M19).

#### Determination of P content

Between 0.5 mg and 10 mg of the samples were weighed into CEM 20-ml MARSXPress PFA vessels with 5 ml of sub-boiled concentrated nitric acid. They were digested using the CEM Plant Material One Touch method (15 min ramp to 200 °C and hold for 15 min) in a MARS6 microwave digestion system. The digested samples were diluted with Milli-Q to 20 ml before being subsampled and further diluted to give a total dilution of approximately 1:60,000. The diluted samples were spiked with Indium and Rhenium to give a concentration of 5 parts per billion to act as internal standards. The samples were analysed in oxygen mode on an Agilent 8900 QQQ-ICP-MS using synthetic standards prepared from Inorganic Ventures ICP-MS single element ICP-MS standards, the standards were also spiked with In and Re at 5 parts per billion (Supplementary Fig. 1, M20).

#### Statistical analyses and reproducibility

All statistical analyses were performed in Sigmaplot 13. To monitor the effects of long-term exposure to nutrient-replete and nutrient-limited conditions, wet weight, relative area, mean grey value data were collected for replicate colonies for each of 10 coral species (10 replicate colonies per parameter and treatment were used for 8 species, 6 replicates for 1 species and 9 replicates for 1 species)^66,67^ (Supplementary Table 1). The replicate colonies were considered technical replicates and their values were averaged for each species. Specific growth responses to nutrient-limited and nutrient-replete conditions returned comparable results for the 10 distinct biological species (Fig. 1).

The growth-enhancing effects of dissolved inorganic N and P (nitrate and phosphate) was reproduced in an entirely different experimental set up used for the ^15^N-labelling experiments (Fig. 2). For these studies, average values for 7–10 replicate colonies per species and treatment were calculated. Host tissue and symbionts of the three distinct biological species showed reproducible responses with regards to the uptake of nitrate and phosphate and the partitioning between the two partners of the symbiosis.

The mechanism of uptake and nutrient distribution was analysed for four biologically distinct species, a subset of the model corals that were also analysed in the experiments for Figs. 1 and 2. The findings were reproducible for all studied species (Fig. 3).

Exponential regressions were used to model the effects of long-term exposure to nutrient-limited and nutrient-replete conditions on the symbiont densities and coral growth (surface area and weight). Linear regressions were used to model the number of digested symbionts against area increase (%) and the ^15^N enrichment between host and symbiont fractions.

Field studies examined samples from nine independent islands across three atolls of the Chagos Archipelago. Replication across the islands of the same type (high versus low seabird densities) revealed reproducible differences that were significantly different for the δ^15^N values of host corals and their symbionts as well as their potential nitrogen sources, zooplankton and bird guano, and nutrient environment-specific growth rates. To compare the ^15^N enrichment between corals (host and symbiont) and their nitrogen sources (zooplankton, macroalgae and guano), the number of biologically independent samples analysed were as follows: (1) for islands with high seabird densities (+Birds): zooplankton (*n* = 18), macroalgae (*n* = 55)^38^, symbionts (*n* = 27), coral host (*n* = 27); and (2) for islands with low seabird densities (−Birds): zooplankton (*n* = 17), macroalgae (*n* = 45), symbionts (*n* = 25), coral host (*n* = 25) (Supplementary Fig. 1, M21).

### Reporting summary

Further information on research design is available in the Nature Portfolio Reporting Summary linked to this article.

## Online content

Any methods, additional references, Nature Portfolio reporting summaries, source data, extended data, supplementary information, acknowledgements, peer review information; details of author contributions and competing interests; and statements of data and code availability are available at 10.1038/s41586-023-06442-5.

## Supplementary information

Supplementary Information This file contains Supplementary Fig. 1 and Supplementary Table 1.

Reporting Summary

## Source data

Source Data Figs. 1–4

## Figures and Tables

**Extended Data Table 1:**
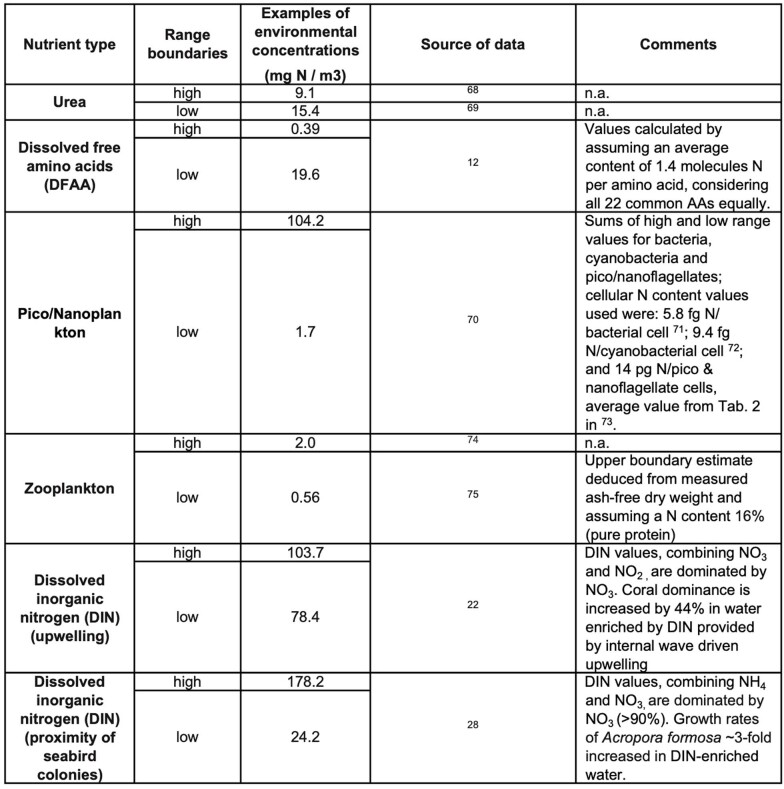
Availability of different types of nitrogen accessible to symbiotic corals

References ^68–75^.

**Extended Data Table 2:**
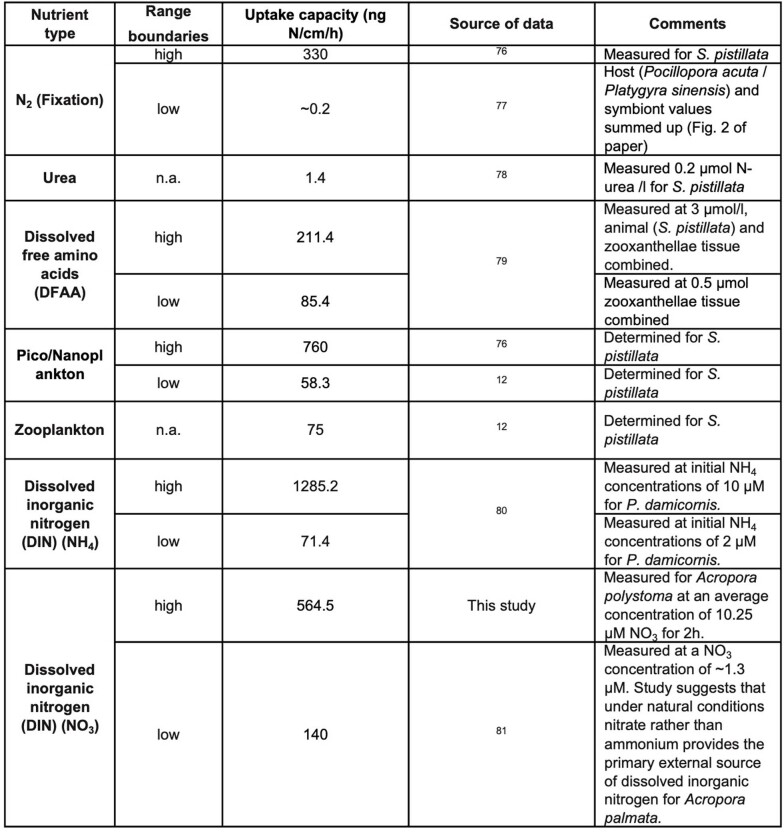
Uptake rates of different types of nitrogen accessible to symbiotic corals

References ^76–81^.

## Data Availability

All data used in this work are available at the University of Southampton data repository subject to standard CC-BY license terms, and can be accessed from 10.5258/SOTON/D2696. Source data are provided with this paper.
