# Author Correction: Reef-building corals farm and feed on their photosynthetic symbionts

**DOI:** 10.1038/s41586-023-06584-6

**Published:** 2023-09-11

**Authors:** Jörg Wiedenmann, Cecilia D’Angelo, M. Loreto Mardones, Shona Moore, Cassandra E. Benkwitt, Nicholas A. J. Graham, Bastian Hambach, Paul A. Wilson, James Vanstone, Gal Eyal, Or Ben-Zvi, Yossi Loya, Amatzia Genin

**Affiliations:** 1 The Coral Reef Laboratory, Ocean and Earth Science, University of Southampton, Southampton, UK; 2 Lancaster Environment Centre, Lancaster University, Lancaster, UK; 3 Ocean and Earth Science, University of Southampton, Southampton, UK; 4 The Mina & Everard Goodman Faculty of Life Sciences, Bar Ilan University, Ramat Gan, Israel; 5 Marine Palaeoecology Laboratory, School of Biological Sciences, The University of Queensland, Brisbane, Queensland Australia; 6 Scripps Institution of Oceanography, University of California, San Diego, La Jolla, CA USA; 7 School of Zoology, The George S. Wise Faculty of Life Sciences, Tel Aviv University, Tel Aviv, Israel; 8 Department of Ecology, Evolution & Behavior, Hebrew University of Jerusalem, Jerusalem, Israel; 9 The Interuniversity Institute for Marine Sciences, Eilat, Israel

**Keywords:** Marine biology, Animal physiology

Correction to: *Nature* 10.1038/s41586-023-06442-5 Published online 23 August 2023

In the version of the article initially published, Gal Eyal’s first affiliation—The Mina & Everard Goodman Faculty of Life Sciences, Bar Ilan University, Ramat Gan, Israel—was missing. This has now been added to the HTML and PDF versions of the article.

