# Supplementary material for: Reef-building corals farm and feed on their photosynthetic symbionts
Source: Nature. 2023 Aug 23;620(7976):1018–24. doi: 10.1038/s41586-023-06442-5 (PMC10468396; doi:10.1038/s41586-023-06442-5)
Supplement: Supplementary file 1 — This file contains Supplementary Fig. 1 and Supplementary Table 1. [file 41586_2023_6442_MOESM1_ESM.pdf]

nature portfolio
https://doi.org/10.1038/s41586-023-06442-5

Supplementary information

Reef-building corals farm and feed on their photosynthetic symbionts

In the format provided by the authors and unedited

Reef-building corals farm and feed on their photosynthetic symbionts

Jörg Wiedenmann, Cecilia D’Angelo, M. Loreto Mardones, Shona Moore, Cassandra E. Benkwitt, Nicholas A.J. Graham, Bastian Hambach, Paul A. Wilson, James Vanstone; Gal Eyal, Or Ben-Zvi, Yossi Loya, Amatzia Genin

Supplementary Information

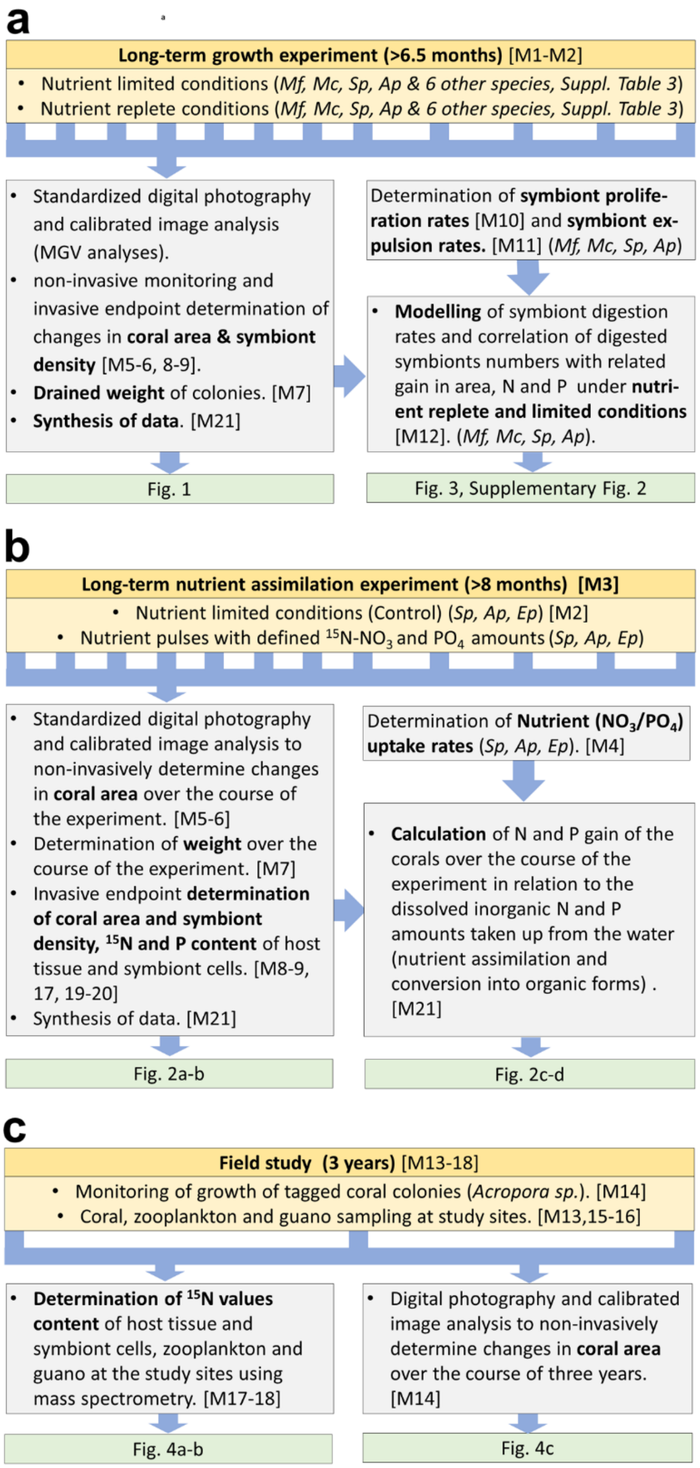

Supplementary Figure 1: Overview of experiments and associated methods. (a) Long-term experiment to determine the growth of corals under replete and limited concentrations of dissolved inorganic N and P in the absence of particulate organic food. (b) Long-term experiment to quantify the uptake and partitioning of 15N-nitrate and phosphate and incorporation as organic N and P in host and symbiont cells in the absence of particulate organic food. (d) Field study to analyse growth and 15N isotopic values and potential nitrogen sources (guano, zooplankton) in corals close to islands with dense seabird colonies and with low seabird numbers. *Mf, Montipora foliosa; Mc, Montipora capricornis; Sp, Stylophora pistillata; Ap, Acropora polystoma; Ep, Euphyllia paradivisa.* Method [M] Numbers in brackets refer to relevant paragraphs in the method section.

Supplementary Table 1: Experimental corals and their symbionts

Experimental coral species	Symbiont species associated with experimental coral (ITS2 sequence) [a]	Growth morphology	Global distribution of coral species [c]
Acropora polystoma	*Cladocopium sp.* (C3cc)<br>(Clade C)	Branching	• 46.7% of global ecoregions<br>• 52.6% of ecoregions in the IndoPacific realm<br>• Red Sea
Montipora capricornis	*Cladocopium sp.* (C26a)<br>(Clade C)	Foliose	• 26% of global ecoregions<br>• 29.3% of ecoregions in the IndoPacific realm
Montipora foliosa	*Cladocopium sp.* (C15)<br>(Clade C)	Foliose	• 58% of global ecoregions<br>• 65.4% of ecoregions in the IndoPacific realm<br>• Red Sea
Porites lichen	*Cladocopium sp.* (C96)<br>(Clade C)	Encrusting	• 68.7% of global ecoregions<br>• 77.4% of ecoregions in the IndoPacific realm<br>• Red Sea & Persian/Arabian Gulf<br>• Eastern Pacific
Seriatopora hystrix	*Durusdinium trenchii / glynnii*<br>(Clade D)	Branching	• 58% of global ecoregions<br>• 65.4% of ecoregions in the IndoPacific realm<br>• Red Sea
Stylophora pistillata	*Symbiodinium microadriaticum*<br>(Clade A)	Branching	• 68.7% of global ecoregions<br>• 77.4% of ecoregions in the IndoPacific realm<br>• Red Sea & Persian/Arabian Gulf
Turbinaria reniformis	*Cladocopium sp.* (C21)<br>*Cladocopium sp.* (C3)<br>(Clade C)	Foliose	• 67.3% of global ecoregions<br>• 75.9% of ecoregions in the IndoPacific realm<br>• Red Sea & Persian/Arabian Gulf
Euphyllia paradivisa	*Cladocopium sp.* (C1)<br>(Clade C) [b]	Phaceloid	• Central Indopacific [d]<br>• Red Sea [e]
Pavona cactus	*n.d.*	Contorted fronds	• 60% of global ecoregions<br>• 67.7% of ecoregions in the IndoPacific realm
Hydnophora grandis	*n.d.*	Encrusting or subarborescent.	• Central Indopacific[d]
Pocillopora damicornis	*n.d.*	Branching	• 85.3% of global ecoregions<br>• 96.2% of ecoregions in the IndoPacific realm<br>• Red Sea & Persian/Arabian Gulf<br>• Eastern Pacific
Lobophytum crassum	*n.d.*	Leather (soft) coral	• Australia, India, Japan, Madagascar, Red Sea, Seychelles, South Africa, Tanzania[d]

[a] From [66]

[b] From [44]

[c] From "Corals of the World – online" (http://www.coralsoftheworld.org/page/home/) unless indicated otherwise

[d] "World Register of Marine Species – online" (https://www.marinespecies.org/index.php)

[e] From [67]
