# Supplementary material for: Reef-building corals farm and feed on their photosynthetic symbionts
Source: Nature. 2023 Aug 23;620(7976):1018–24. doi: 10.1038/s41586-023-06442-5 (PMC10468396; doi:10.1038/s41586-023-06442-5)
Supplement: Supplementary file 2 — Reporting Summary [file 41586_2023_6442_MOESM2_ESM.pdf]

nature portfolio

Corresponding author(s): Joerg Wiedenmann

Last updated by author(s): 29/06/2023

Reporting Summary

Nature Portfolio wishes to improve the reproducibility of the work that we publish. This form provides structure for consistency and transparency in reporting. For further information on Nature Portfolio policies, see our Editorial Policies and the Editorial Policy Checklist.

Statistics

For all statistical analyses, confirm that the following items are present in the figure legend, table legend, main text, or Methods section.

n/a	Confirmed	
☐	☒	The exact sample size (*n*) for each experimental group/condition, given as a discrete number and unit of measurement
☐	☒	A statement on whether measurements were taken from distinct samples or whether the same sample was measured repeatedly
☐	☒	The statistical test(s) used AND whether they are one- or two-sided<br>*Only common tests should be described solely by name; describe more complex techniques in the Methods section.*
☒	☐	A description of all covariates tested
☒	☐	A description of any assumptions or corrections, such as tests of normality and adjustment for multiple comparisons
☐	☒	A full description of the statistical parameters including central tendency (e.g. means) or other basic estimates (e.g. regression coefficient) AND variation (e.g. standard deviation) or associated estimates of uncertainty (e.g. confidence intervals)
☐	☒	For null hypothesis testing, the test statistic (e.g. F, t, r) with confidence intervals, effect sizes, degrees of freedom and P value noted<br>*Give P values as exact values whenever suitable.*
☒	☐	For Bayesian analysis, information on the choice of priors and Markov chain Monte Carlo settings
☒	☐	For hierarchical and complex designs, identification of the appropriate level for tests and full reporting of outcomes
☒	☐	Estimates of effect sizes (e.g. Cohen's *d*, Pearson's *r*), indicating how they were calculated

Our web collection on statistics for biologists contains articles on many of the points above.

Software and code

Policy information about availability of computer code

Data collection	no software was used for data collection
Data analysis	ImageJ/Fiji; Sigma Plot

For manuscripts utilizing custom algorithms or software that are central to the research but not yet described in published literature, software must be made available to editors and reviewers. We strongly encourage code deposition in a community repository (e.g. GitHub). See the Nature Portfolio guidelines for submitting code & software for further information.

Data

Policy information about availability of data

All manuscripts must include a data availability statement. This statement should provide the following information, where applicable:

- Accession codes, unique identifiers, or web links for publicly available datasets
- A description of any restrictions on data availability
- For clinical datasets or third party data, please ensure that the statement adheres to our policy

All Data used in this work is available at the University of Southampton data repository, subject to standard CC-BY license terms, and can be accessed through the link: https://doi.org/10.5258/SOTON/D2696

Human research participants

Policy information about studies involving human research participants and Sex and Gender in Research.

Reporting on sex and gender	n.a.
Population characteristics	n.a.
Recruitment	n.a.
Ethics oversight	n.a.

Note that full information on the approval of the study protocol must also be provided in the manuscript.

Field-specific reporting

Please select the one below that is the best fit for your research. If you are not sure, read the appropriate sections before making your selection.

☐ Life sciences ☐ Behavioural & social sciences ☒ Ecological, evolutionary & environmental sciences

For a reference copy of the document with all sections, see nature.com/documents/nr-reporting-summary-flat.pdf

Ecological, evolutionary & environmental sciences study design

All studies must disclose on these points even when the disclosure is negative.

Study description	1) Controlled Laboratory experiments involving two different nutrient conditions. Data were collected over time (203 days) and assembled in a time course. In each condition up to 10 replicate colonies for each of the 10 species under study were exposed to the experimental treatment.
Research sample	A set of cosmopolitan or widely distributed host coral species representing different growth morphologies (branching, foliose, encrusting) to ensure that the findings are representative. Molecular sequencing confirmed that 8 symbiont taxa were covered, including divergent species such as Cladocopium and Durusdinium.
Sampling strategy	As an overall strategy, we used a randomized design with species as biological replicates (10), underpinned by technical replicates (up to 10).
Data collection	Data were collected throughout the experiments in regular intervals at the same time of the day. Designed and conducted aquarium experiments (JW, CDA, MLM, SM), Analysed symbiont taxonomy (JV and CDA), Designed and conducted field studies (CB and NG with input of JW and CDA), Designed and conducted stable isotope analyses (MLM, BH, PAW with input from JW and CDA).
Timing and spatial scale	Experiments were performed during 2019 to 2021. The stable isotope labeling experiment ran from 15 August 2019 for 222 days. The multi-species experiments started on 5th January 2021 and ran for 203 days.
Data exclusions	Not applicable
Reproducibility	The stable isotope labeling experiment and the multi-species experiments were following a similar overall design, but ran in different experimental tanks and at non-overlapping times. The outcomes of both longterm experiments support the same conclusion. The mathematical model used to quantify symbiont digestion rates were applied to corals from completely different condition (nutrient replete vs nutrient limited), still the model could describe the in-vivo observations with high precision.
Randomization	Randomised design was used to distribute samples across different treatments.
Blinding	The nature of the physiological measurements require the precise identification of the individual sample for correct allocation of the measurement values of the individual within the time course of recording.

Did the study involve field work? ☒ Yes ☐ No

Field work, collection and transport

Field conditions	In May 2018, branching Acropora sp. colonies were sampled from 9 uninhabited islands in the northern atolls of the Chagos Archipelago, Indian Ocean. Five of the islands had diverse and abundant seabird populations which provide substantial nutrient subsidies to the nearshore marine environment, while 4 of the islands had few seabirds due to the presence of introduced rats.

Location	Northern atolls of the Chagos Archipelago, Indian Ocean. British Indian Ocean Territory (-6.682094, 71.358606)
Access & import/export	Fieldwork was conducted under permit numbers 0004SE18, 0001SE19, 0003SE20, and 0002SE21 and related import permits.
Disturbance	All sampling sites are uninhabited by humans, Sample sizes were reduced to the minimal size sufficient to conduct the analyses. Only fragments of colonies were removed so that these highly regenerative organisms can continue to grow with out any losses from the genepool.

Reporting for specific materials, systems and methods

We require information from authors about some types of materials, experimental systems and methods used in many studies. Here, indicate whether each material, system or method listed is relevant to your study. If you are not sure if a list item applies to your research, read the appropriate section before selecting a response.

Materials & experimental systems

n/a	Involved in the study	
☒	☐	Antibodies
☒	☐	Eukaryotic cell lines
☒	☐	Palaeontology and archaeology
☐	☒	Animals and other organisms
☒	☐	Clinical data
☒	☐	Dual use research of concern

Methods

n/a	Involved in the study	
☒	☐	ChIP-seq
☒	☐	Flow cytometry
☒	☐	MRI-based neuroimaging

Animals and other research organisms

Policy information about studies involving animals; ARRIVE guidelines recommended for reporting animal research, and Sex and Gender in Research

Laboratory animals	Acropora polystoma<br>Montipora capricornis<br>Montipora foliosa<br>Porites lichen<br>Seriatopora hystrix<br>Stylophora pistillata<br>Turbinaria reniformis<br>Euphyllia paradivisa<br>Pavona cactus<br>Hydnophora grandis<br>Pocillopora damicornis<br>Lobophytum crassum<br><br>All corals (non-vertebrate organisms) are grown in our laboratory since 2008 or longer. All experimental samples were produced through in-house propagation. Accordingly, the experimental approach is entirely sustainable.
Wild animals	Samples from wild corals (Acropora sp.) were obtained by removing small fragments from the colony and freezing them immediately on arrival back on the boat.
Reporting on sex	Corals are often hermaphrodites, accordingly the matter of sex is not applicable in the context.
Field-collected samples	No field collected samples were kept alive.
Ethics oversight	Ethics are controlled by ERGO II (Ethics and Research Governance Online) hosted by the University of Southampton.

Note that full information on the approval of the study protocol must also be provided in the manuscript.
